# Synergistic Anti-Cancer Effects of ERB-041 and Genistein through Estrogen Receptor Suppression-Mediated PI3K/AKT Pathway Downregulation in Canine Mammary Gland Tumor Cells

**DOI:** 10.3390/ijms25052466

**Published:** 2024-02-20

**Authors:** Min-Jae Yoo, Ye-Ji Jang, Sang-Youel Park, Ja-Wun Choi, Jae-Won Seol

**Affiliations:** College of Veterinary Medicine, Jeonbuk National University, Iksan 54596, Jeollabuk-do, Republic of Korea; ymin105@naver.com (M.-J.Y.); yejiown25@gmail.com (Y.-J.J.); sypark@chonbuk.ac.kr (S.-Y.P.)

**Keywords:** canine-mammary-gland tumors, estrogen receptor, ERB-041, genistein, PI3K/AKT pathway

## Abstract

Canine-mammary-gland tumors (CMTs) are prevalent in female dogs, with approximately 50% of them being malignant and often presenting as inoperable owing to their size or metastasis. Owing to poor outcomes, effective alternatives to conventional chemotherapy for humans are necessary. Two estrogen receptors, estrogen receptor alpha (ERα) and estrogen receptor beta (ERβ), which act in opposition to each other, are involved, and CMT growth involves ERα through the phosphoinositide 3-kinases (PI3K)/AKT pathway. In this study, we aimed to identify the synergistic anti-cancer effects of ERB-041, an ERβ agonist, and genistein, an isoflavonoid from soybeans known to have ERβ-specific pseudo-estrogenic actions, on CMT-U27 and CF41.Mg CMT cell lines. ERB-041 and genistein synergistically inhibited cell proliferation and increased the number of annexin V-positive cells in both cell lines. Furthermore, we observed a synergistic increase in the Bax/Bcl-2 ratio and cleaved caspase-3 expression. Additionally, cell-cycle arrest occurred through the synergistic regulation of cyclin D1 and cyclin-dependent kinase 4 (CDK4). We also found a synergistic decrease in the expression of ERα, and the expression of proteins involved in the PI3K/AKT pathway, including p-PI3K, phosphatase and tensin homolog (PTEN), AKT, and mechanistic target of rapamycin (mTOR). In conclusion, ERB-041 and genistein exhibited a synergistic anticancer effect on CMTs, suggesting that cotreatment with ERB-041 and genistein is a promising treatment for CMTs.

## 1. Introduction

Canine-mammary-gland tumors (CMTs) are the most common tumors in female dogs and approximately 50% of these tumors are malignant [1]. Approximately 50% of these tumors are inoperable because they are excessively large or metastasized; therefore, chemotherapy is administered [2]. The chemotherapeutic agents used in these cases are designed for humans and do not have a significant therapeutic effect in the treatment of CMTs [3]. In recent years, several methods have been used to treat CMTs, including surgery, radiotherapy, chemotherapy, and hormonal therapy. Chemotherapy is frequently used for CMTs that have metastasized or have a high probability of recurrence; however, the effectiveness of chemotherapeutic agents has not been proven, leading to high recurrence rates and poor prognosis [4,5]. Therefore, it is necessary to identify effective chemotherapeutic agents for the treatment of CMTs.

Estrogen is a steroid hormone that acts as a primary female sex hormone and affects many aspects of the body, including growth and differentiation. There are three main forms of estrogen: estrone (E1), estradiol (E2), and estriol (E3) [6]. These molecules primarily interact with two types of estrogen receptors, estrogen receptor alpha (ERα) and estrogen receptor beta (ERβ), to perform their roles. ER, a ligand-dependent transcription factor, regulates gene transcription through estrogen response elements, thereby promoting the normal biological functions of estrogen [7]. In numerous cases of breast cancer, the increased proliferation is commonly attributed to the activation of ERα by estrogen, which is counterbalanced by the existence of ERβ, which imparts an inhibitory effect on cell growth [8]. In addition, previous studies have reported that high ERβ levels reduce ERα expression [9]. In contrast to the way normal mammary glands express both ERα and ERβ positively, CMTs have been reported to exhibit a predominantly ERβ-positive status and a weaker intensity of ERα [10]. Despite these ERβ-dominant features, CMTs are fatal with low survival rates [11].

The phosphoinositide 3-kinase (PI3K)/AKT pathway, which consists of PI3K, a phosphatase and tensin homolog (PTEN), AKT (also known as protein kinase B), and a mechanistic target of rapamycin (mTOR), is one of the most important pathways involved in cell death, metabolism, proliferation, and cell-cycle regulation [12]. In addition, PI3K, a key enzyme in this pathway, is considered an important therapeutic target because its gene *PI3KCA* is highly mutated in many tumors, including human breast cancer and CMTs, increasing the expression of downstream signals and promoting tumor growth [13,14]. Furthermore, in breast cancers, estrogen activates the PI3K/AKT pathway through ERα to increase downstream signaling, leading to tumor growth, invasion, and metastasis [15]. Therefore, it is essential to confirm whether the ERα inhibition effect occurs when treated with an ERβ agonist. If this inhibitory effect leads to the inhibition of the PI3K/AKT pathway, it may contribute to the treatment of CMTs.

ERB-041 is a potent and selective ERβ agonist and has been reported to exhibit anti-inflammatory effects in Phase II clinical trials for the treatment of rheumatoid arthritis [16,17,18]. ERB-041 inhibits triple-negative breast-cancer-cell invasion [19]. Furthermore, ERB-041 decreased cell migration, invasion, and proliferation and induced cell-cycle arrest by modulating the PI3K/AKT pathway in ovarian cancer cells [20]. Genistein is a phytoestrogen that belongs to the flavonoid family and is derived from soybeans and soy-derived foods [21]. Genistein has been reported to have anti-cancer effects in breast cancers, demonstrating its potential as a therapeutic agent [22]. Additionally, it inhibits the PI3K/AKT pathway in triple-negative breast cancer cells [23]. The most important characteristic of genistein is that as a phytoestrogen, it binds to and acts more specifically on ERβ than ERα [24]. Both ERB-041 and genistein act as ERβ agonists, showing selectivity with differences in the ligands that bind to ERβ [25,26]. Therefore, the aim of the present study was to determine whether two agonists can have a synergistic anticancer effect on CMTs.

## 2. Results

### 2.1. ERB-041 and Genistein Synergistically Inhibit Proliferation of CMT Cells

**Figure 1:**
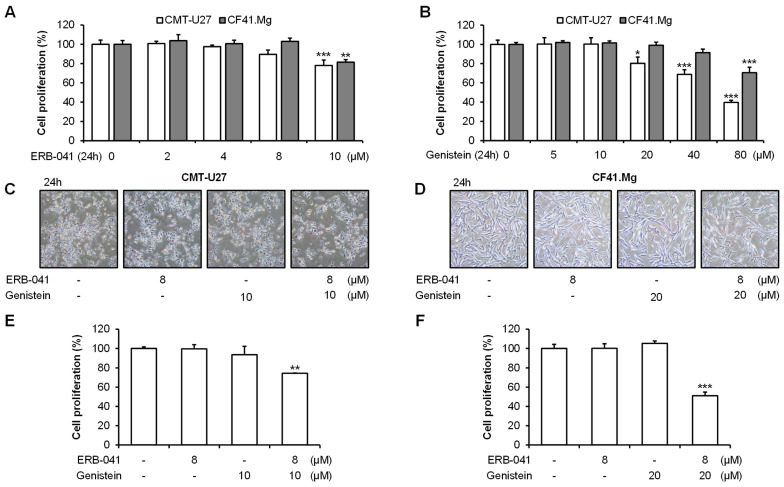
ERB-041 and genistein synergistically suppress the proliferation of CMT cells. (**A**,**B**) Cell viability at indicated concentrations of ERB-041 and genistein in CMT-U27 (**A**) and CF41.Mg (**B**); (**C**,**D**) morphological images of CMT-U27 (**C**) and CF41.Mg (**D**); (**E**,**F**) synergistic cell viability reduction in CMT-U27 (**E**) and CF41.Mg (**F**) following treatment with ERB-041 and genistein at the indicated concentrations. Magnification, 100×. Values are mean ± SD. * *p* < 0.05; ** *p* < 0.01; *** *p* < 0.001 versus untreated cells by one-way ANOVA followed by Bonferroni post hoc test.

To confirm the effective concentrations of ERB-041 and genistein against two CMT cell lines, a 3-(4,5-dimethylthiazol-2-yl)-5-(3-carboxymethoxyphenyl)-2-(4-sulfophenyl)-2H-tetrazolium (MTS) assay was performed on the CMT-U27 and CF41.Mg cell lines according to each determined concentration. At 10 μM, ERB-041 induced significant proliferation inhibition in both cell lines, whereas genistein induced significant proliferation inhibition at 20 μM in CMT-U27 and 80 μM in CF41.Mg cells (Figure 1A,B). It is important to consider the fact that CMT-U27 has the characteristics of an epithelial cell and CF41.Mg has the characteristics of a mesenchymal cell, which means that the effective concentration of genistein for each is different. Therefore, we examined the synergistic effect of 8 μM ERB-041 and 10 μM genistein in CMT-U27 and 8 μM ERB-041 and 20 μM genistein in CF41.Mg cells, as concentrations that did not significantly reduce cell viability. We observed the morphology of CMT-U27 and CF41.Mg cells and performed an MTS assay. Morphological imaging of both cell lines showed that the combination of drugs synergistically reduced the number of cells and altered their morphology (Figure 1C,D). In the MTS assay, the treatment of CMT-U27 cells with ERB-041 and genistein inhibited cell proliferation by approximately 1.4% and 6.3%, respectively, compared to the control group, showing no significant difference. However, upon cotreatment, a significant synergistic proliferation-inhibition effect of approximately 25.5% compared with the control group was observed (Figure 1E). Similarly, compared to the control group, the proliferation of CF41.Mg cells did not significantly differ with ERB-041 and genistein treatment. However, with cotreatment, a significant inhibitory effect of approximately 48.9% compared with that in the control group was observed (Figure 1F). These results indicate that ERB-041 and genistein synergistically inhibited cell proliferation in both CMT-U27 and CF41.Mg cell lines.

### 2.2. ERB-041 and Genistein Synergistically Induce Apoptosis in CMT Cells by Regulating the Expression of Bcl-2/Bax/Caspase-3

**Figure 2:**
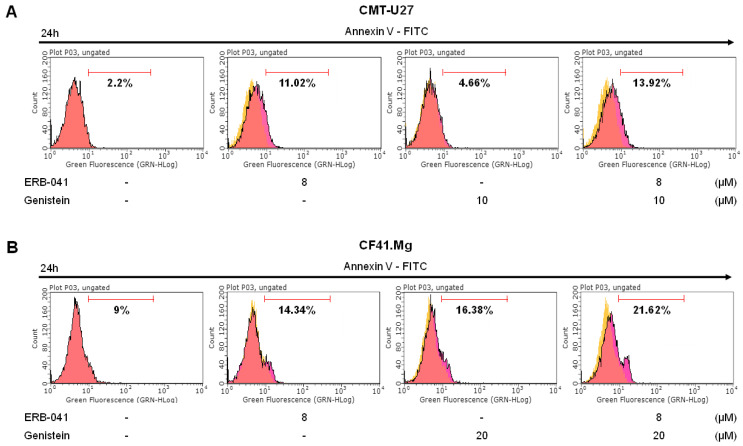
ERB-041 and genistein synergistically induced apoptosis in CMT cells. (**A**,**B**) Images of Annexin-V/PI staining results of CMT-U27 (**A**) and CF41.Mg (**B**) cells treated with ERB-041 and genistein at the indicated concentrations.

**Figure 3:**
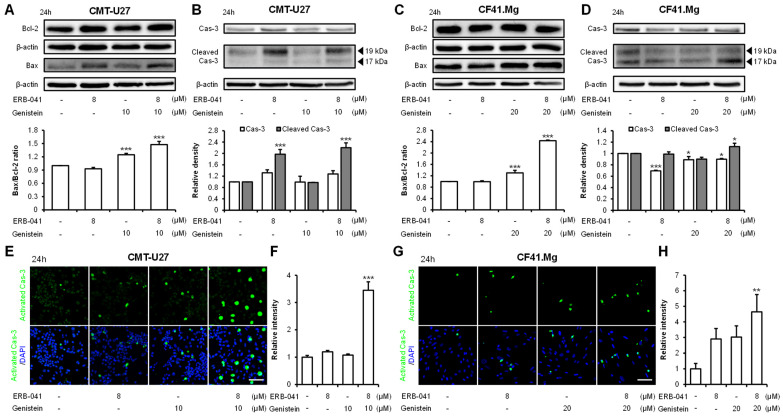
ERB-041 and genistein are involved in the regulation of Bcl-2, Bax, and caspase-3. (**A**,**B**) Western blot images and quantification of the expression of Bcl-2, Bax (**A**), caspase-3, and cleaved caspase-3 (**B**) in CMT-U27 upon ERB-041 and genistein treatment at the indicated concentrations and the expression of Bcl-2, Bax (**C**), caspase-3, and cleaved caspase-3 (**D**) in CF41.Mg.; (**E**–**H**) immunocytochemistry images and quantitative analysis of activated caspase-3 in CMT-U27 (**E**,**F**) and CF41.Mg (**G**,**H**). Cas-3, caspase-3. Scale bar, 50 µm. Values are mean ± SD. * *p* < 0.05; ** *p* < 0.01; *** *p* < 0.001 versus untreated cells by one-way ANOVA followed by Bonferroni post hoc test.

To determine the effects of ERB-041 and genistein on CMT cell death, we performed an Annexin-V/Propidium Iodide (PI) assay. The results showed that in CMT-U27 cells, ERB-041 treatment increased cell death by approximately 5-fold compared to that in the control group, and genistein treatment increased cell death by approximately 2-fold. In addition, the combination treatment induced approximately 6.5-fold more cell death than the control (Figure 2A). Similarly, in CF41.Mg cells, ERB-041 and genistein increased cell death by approximately 1.6- and 1.8-fold, respectively, compared to that in the control group, and by approximately 2.4-fold when combined compared to that in the control group (Figure 2B). To confirm apoptosis induction, Western blotting was performed to determine the expression of Bcl-2, Bax, caspase-3, and cleaved caspase-3. The Bax/Bcl-2 ratio in CMT-U27 cells was not significant when treated with ERB-041 alone but increased significantly to approximately 25% compared to that in the control group when treated with genistein alone and to approximately 48% when treated with both agonists (Figure 3A and Figure S1). Caspase-3 expression did not differ significantly between the treatment and control groups. In contrast, cleaved caspase-3 expression showed no difference when treated with genistein alone, but showed a significant increase of approximately 98% when treated with ERB-041 and an increase of approximately 120% when cotreated compared to that in the control group (Figure 3B and Figure S1). Protein expression in the CF41.Mg cells was similar to that in the Western blot-treated CMT-U27 cells. In CF41.Mg, a significant difference was absent in the Bax/Bcl-2 ratio when treated with ERB-041 alone. However, when treated with genistein alone or cotreated, the Bax/Bcl-2 ratio increased by approximately 30% and 143%, respectively, compared to that in the control group, which was highly significant (Figure 3C and Figure S2). Caspase-3 expression was reduced by approximately 31%, 11%, and 10% compared with that in the control group when treated with ERB-041, genistein, or both, respectively, and the expression of cleaved caspase-3 was reduced by approximately 1%, 10%, and 12%, respectively, compared with that in the control group (Figure 3D and Figure S2). Activated caspase-3 was identified using immunocytochemistry. A significant difference was absent in the expression of activated caspase-3 between the ERB-041 and genistein alone groups. However, with cotreatment, CMT-U27 and CF41.Mg showed significant increases of approximately 245% and 34%, respectively, compared to that in the control group (Figure 3E–H). These results indicate that ERB-041 and genistein synergistically induced apoptosis in both CMT-U27 and CF41.Mg cell lines.

### 2.3. ERB-041 and Genistein Synergistically Induce Cell-Cycle Arrest by Regulating Cell-Cycle-Related Proteins in CMT Cells

**Figure 4:**
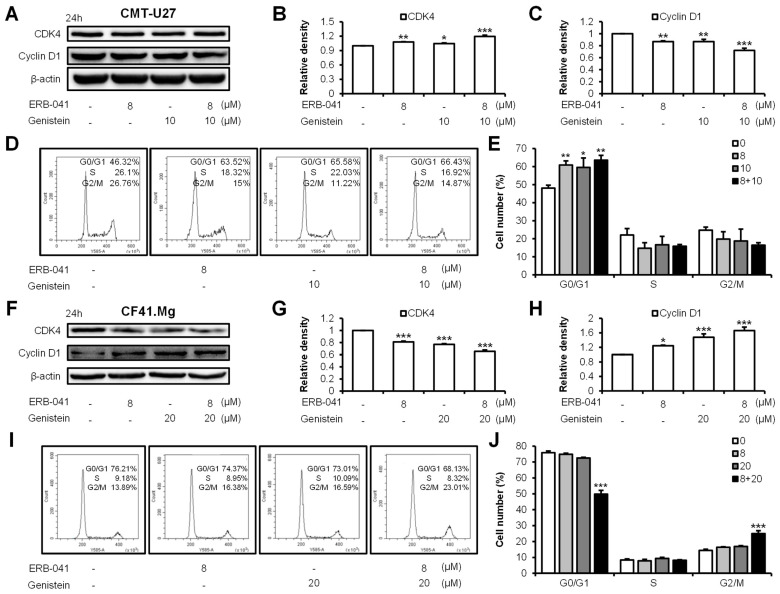
ERB-041 and genistein induce cell-cycle arrest in CMT cells through synergistic regulation of CDK4 and cyclin D1. (**A**–**C**) Western blotting images and quantification of CDK4 (**A**,**B**) and cyclin D1 (**A**,**C**) following treatment with indicated concentrations of ERB-041 and genistein in CMT-U27; (**D**,**E**) cell-cycle-arrest assay data by flow cytometry (**D**) and quantitative analysis (**E**) in CMT-U27; (**F**–**H**) Western blotting images and quantification of CDK4 (**F**,**G**) and cyclin D1 (**F**,**H**) after treatment with ERB-041 and genistein at the concentrations indicated in CF41.Mg; (**I**,**J**) cell-cycle-arrest assay data by flow cytometry (**I**) and quantitative analysis (**J**) in CF41.Mg. CDK4, cyclin-dependent kinase 4. Values are mean ± SD. * *p* < 0.05; ** *p* < 0.01; *** *p* < 0.001 versus untreated cells by one-way ANOVA followed by Bonferroni post hoc test.

To investigate the effects of ERB-041 and genistein on the cell cycle of CMT-U27 and CF41.Mg, we performed Western blotting with anti-CDK4 and -cyclin D1 antibodies and a cell-cycle arrest assay (Figure 4A–E and Figure S3). When CMT-U27 cells were treated with ERB-041, genistein, and both, the expression of CDK4 increased by approximately 8%, 5%, and 19%, respectively, compared to that of the control group. In contrast to the expression of CDK4, cyclin D1 expression was reduced by approximately 13%, 13%, and 28% in the three groups, respectively, compared to that of the control group. Subsequently, a cell-cycle arrest assay revealed a G0/G1 phase arrest. The number of cells in the G0/G1 phase increased synergistically from approximately 48% in the control to 61% with ERB-041 treatment alone, 60% with genistein treatment alone, and 64% with cotreatment. A Western blotting and cell-cycle arrest assay in CF41.Mg cells showed contrasting effects to that in CMT-U27 cells (Figure 4F–J and Figure S3). The expression of CDK4 in the CF41.Mg cells decreased by approximately 19%, 23%, and 35% compared to that in the control group when treated with ERB-041, genistein, or both, respectively. Conversely, the expression of cyclin D1 increased by approximately 24%, 48%, and 66%, respectively, in each group compared to that in the control group. In the cell-cycle arrest assay, a significant difference in cell number was observed only in the cotreated group with ERB-041 and genistein. The number of cells in the G0/G1 phase decreased by approximately 26% in the cotreatment group compared to the control group, and the number of cells in the G2/M phase increased by approximately 11% compared to that in the control group, indicating G2/M phase arrest. These results indicate that ERB-041 and genistein synergistically regulate the expression of cell-cycle-related proteins to induce cell-cycle arrest in CMT cells.

### 2.4. ERB-041 and Genistein Synergistically Inhibit the Expression of ERα

**Figure 5:**
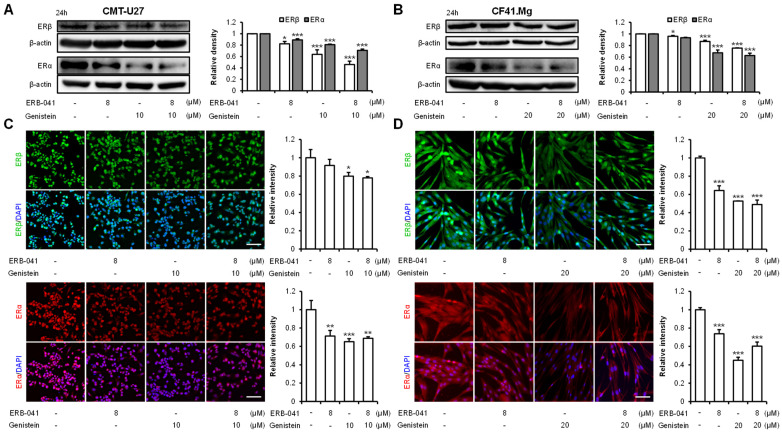
Effects of ERB-041 and genistein treatment on the expression of ERβ and ERα. (**A**,**B**) Western blot images and quantitative analysis of ERβ and ERα upon treatment with ERB-041 and genistein at the concentrations indicated in CMT-U27 (**A**) and CF41.Mg (**B**); (**C**,**D**) immunocytochemistry images and quantification of ERβ and ERα in CMT-U27 (**C**) and CF41.Mg (**D**). Scale bar, 50 µm. ERβ, estrogen receptor beta; ERα, estrogen receptor alpha. Values are mean ± SD. * *p* < 0.05; ** *p* < 0.01; *** *p* < 0.001 versus untreated cells by one-way ANOVA followed by Bonferroni post hoc test.

Western blot analysis was conducted to assess the alterations in the expression of ERα and ERβ upon treatment with ERB-041 and genistein, which both function as ERβ agonists. Upon treatment with ERB-041, genistein, and their combination, in CMT-U27 cells, the expression of ERβ exhibited significant reductions by approximately 18%, 36%, and 54%, respectively, compared to that in the control group. Similarly, the expression of ERα showed substantial decreases of approximately 11%, 19%, and 29% compared to that in the control group upon treatment with ERB-041, genistein, and cotreatment, respectively (Figure 5A and Figure S4). CF41.Mg cells showed similar results, with ERB-041 alone, genistein alone, and combined treatment reducing ERβ expression by approximately 4%, 13%, and 24%, respectively, compared to that of the control group. The expression of ERα was not significantly different from that of the control group in the ERB-041 treated group, whereas it was significantly reduced by approximately 32% and 37% in the genistein and cotreated groups, respectively, compared to that in the control group (Figure 5B and Figure S4). Immunocytochemistry was then performed to confirm the Western blotting results for ERβ and ERα. The expression of ERβ in CMT-U27 was reduced by approximately 8%, 20%, and 22% when treated with ERB-041 alone, genistein alone, and both treatments together, respectively, compared to that in the control group. ERα expression was significantly different in the three groups, showing a decrease of approximately 29%, 35%, and 31%, respectively, compared to that in the control group (Figure 5C). CF41.Mg cells showed the same results, with ERβ expression significantly declining by approximately 36%, 47%, and 51% compared to that in the control group when treated with ERB-041 alone, genistein alone, or together, respectively. The expression of ERα was greatly reduced in the three groups to approximately 26%, 55%, and 40%, respectively, compared to that in the control group. The most significant decrease occurred in the group treated with genistein alone. However, all three groups showed substantial differences (Figure 5D). These results suggest that ERα is repressed by the ERβ agonists ERB-041 and genistein in both CMT cell lines.

### 2.5. ERB-041 and Genistein Synergistically Inhibit the Expression of the PI3K/AKT Pathway-Related Proteins

**Figure 6:**
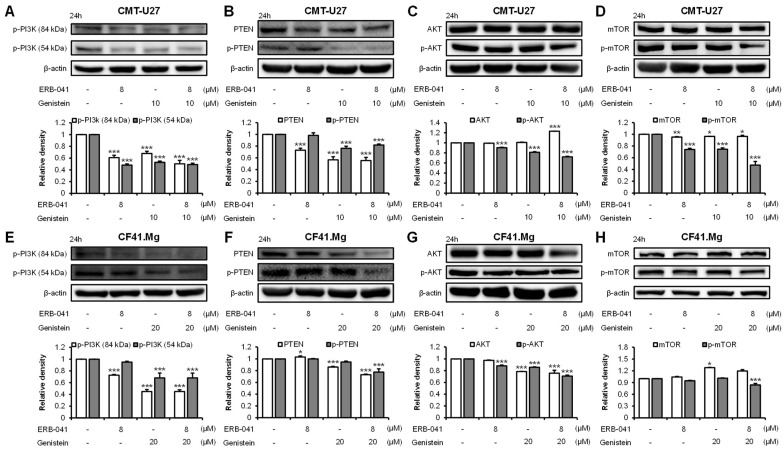
Western blotting results demonstrate that ERB-041 and genistein synergistically regulate the expression of the PI3K/AKT pathway. (**A**–**D**) Western blot images and quantification of p-PI3K (**A**), PTEN, p-PTEN (**B**), AKT, p-AKT (**C**), mTOR, and p-mTOR (**D**) expression in CMT-U27 following ERB-041 and genistein treatment; (**E**–**H**) Western blot images and quantification of the expression of PI3K/AKT-pathway-related proteins in CF41.Mg of ERB-041 and genistein at the indicated concentrations. PI3K, phosphoinositide 3-kinases; PTEN, phosphatase and tensin homolog; mTOR, mechanistic target of rapamycin. Values are mean ± SD. * *p* < 0.05; ** *p* < 0.01; *** *p* < 0.001 versus untreated cells by one-way ANOVA followed by Bonferroni post hoc test.

**Figure 7:**
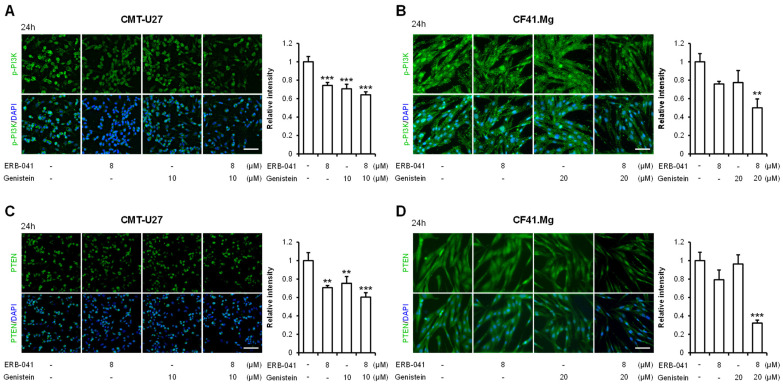
Immunocytochemistry results demonstrate that ERB-041 and genistein synergistically reduce the expression of the PI3K and PTEN. (**A**–**D**) Immunocytochemistry images and quantitative analysis of the p-PI3K expression in CMT-U27 (**A**) and CF41.Mg (**B**) when treated with ERB-041 and genistein at the indicated concentrations and PTEN expression in CMT-U27 (**C**) and CF41.Mg (**D**). PI3K, phosphoinositide 3-kinases; PTEN, phosphatase and tensin homolog. Scale bar, 50 µm. Values are mean ± SD. ** *p* < 0.01; *** *p* < 0.001 versus untreated cells by one-way ANOVA followed by Bonferroni post hoc test.

To confirm that the previously identified inhibition of ERα expression in CMT cells by ERB-041 and genistein leads to the inhibition of the PI3K/AKT pathway, Western blotting was performed. In CMT-U27, Western blot analysis showed that the protein expression levels of p-PI3K, PTEN, p-AKT, and p-mTOR were reduced. The p-PI3K level was reduced by approximately 39% and 52% with ERB-041 treatment alone (84 and 54 kDa), 32% and 47% with genistein treatment alone, and 50% and 51% with their cotreatment, respectively, compared to that in the control group (Figure 6A and Figure S5). The expression of PTEN was reduced by approximately 27% compared to that in the control group when treated with ERB-041 alone, 43% when treated with genistein alone, and 45% when treated together; the expression of p-PTEN was decreased by 2%, 24%, and 18%, respectively, compared to that in the control group (Figure 6B and Figure S5). AKT expression was only significant in the cotreatment group, increasing by approximately 23% compared with that in the control group. In contrast, the expression of p-AKT decreased by approximately 10% compared to that in the control group with ERB-041 alone, by approximately 19% with genistein alone, and by approximately 28% with cotreatment, with the decrease being significant in all groups (Figure 6C and Figure S5). Furthermore, the expression of mTOR was reduced by approximately 5%, 3%, and 3% compared to that in the control group when cells were treated with ERB-041 alone, genistein alone, and both, respectively, and p-mTOR expression was decreased by approximately 26%, 25%, and 53%, respectively, compared to that in the control group (Figure 6D and Figure S6). In CF41.Mg cells, a similar pattern of protein expression to that observed in CMT-U27 was observed with Western blotting. The expression of p-PI3K was significantly reduced by 27% and 5% when treated with ERB-041 alone (84 and 54 kDa) and by 55% and 32% when treated with genistein alone and together, respectively, compared to that in the control (Figure 6E and Figure S6). The expression of PTEN increased by approximately 3% compared to the control group when treated with ERB-041 alone, decreased by approximately 14% when treated with genistein alone, and by approximately 27% when treated with both, compared to that in the control group. The expression of p-PTEN declined by approximately 22% compared to that in the control group control when cotreated, which was the only significant finding (Figure 6F and Figure S6). The expression of AKT decreased by approximately 21% compared to that in the control group when treated with genistein alone and by approximately 24% when cotreated with ERB-041 and genistein, whereas the expression of p-AKT was significantly reduced by approximately 12%, 14%, and 29% compared to that in the control group when treated with ERB-041 alone, genistein alone, and cotreated, respectively (Figure 6G and Figure S7). The expression of mTOR increased by approximately 4%, 28%, and 19% when treated with ERB-041 alone, genistein alone, and their combination, respectively, compared to that in the control group. Furthermore, p-mTOR expression was reduced by approximately 16% in the cotreatment group compared to that in the control group, which was highly significant (Figure 6H and Figure S7). We then performed immunocytochemistry to confirm the synergistic effect of ERB-041 and genistein on the inhibition of the PI3K/AKT pathway, as observed through Western blotting. The results showed that the expression of p-PI3K in CMT-U27 cells showed a significant synergistic decrease, with reductions of approximately 26%, 29%, and 36% compared to that in the control group upon treatment with ERB-041 alone, genistein alone, and their cotreatment, respectively (Figure 7A). The expression of PTEN also exhibited a synergistic effect, showing reductions of approximately 28%, 25%, and 40%, respectively, in the three treatment groups compared to that in the control group (Figure 7C). The expression of p-PI3K in CF41.Mg cells was reduced by approximately 24% compared to that in the control group when treated with ERB-041 alone, 23% when treated with genistein alone, and approximately 50% when treated with cotreatment, with significance only in the cotreatment group (Figure 7B). In addition, PTEN expression was reduced by approximately 21%, 4%, and 68% in the three treatment groups compared to that in the control group, with significance only in the cotreatment group (Figure 7D). These results indicated that ERB-041 and genistein synergistically inhibited the expression of PI3K/AKT pathway-related proteins in both CMT cell lines.

## 3. Discussion

In numerous cases of human breast cancer, growth is commonly promoted by estrogen-activated ERα, which is counterbalanced by the cell growth-inhibitory effects of ERβ [27]. Similarly, in veterinary research, ERα activated by E2 has been demonstrated to play an important role in the growth of CMTs [28]. In addition, the development of CMTs is estradiol-dependent, wherein many of these patients express high tissue levels of ERα or elevated concentrations of serum E2 [28,29]. However, the selective estrogen receptor modulators such as tamoxifen and raloxifene, which act as antagonists to ERα, did not show a significant effect on CMTs, probably owing to a low affinity for the receptor [10]. Furthermore, the selective activation of ERα or ERβ can be influenced not only by the affinity for selective receptor binding but also by the selective activation of each receptor subtype [30]. In this study, we aimed to determine whether treatment with ERB-041 and genistein, which act as ERβ agonists, can exert anti-cancer effects on CMTs by reducing the expression of ERα. Our results demonstrate that ERB-041 and genistein synergistically decreased ERα expression, resulting in PI3K/AKT pathway inhibition and the induction of cell apoptosis and cell-cycle arrest in both CMT-U27 and CF41.Mg cell lines.

Numerous studies suggest that the induction of apoptosis and cell-cycle arrest are major strategies in anti-cancer therapy [31,32]. Previous studies have shown that ERB-041 and genistein increase the Bax/Bcl-2 ratio and expression of cleaved caspase-3, a marker of apoptosis induction in various human cancers [33,34]. Our findings indicate that ERB-041 and genistein synergistically inhibited the proliferation of CMT-U27 and CF41.Mg cells. In addition, Annexin-V/PI staining demonstrated a synergistic increase in apoptosis when both cell lines were treated with ERB-041 and genistein. Moreover, synergistic effects were identified through the ratio of Bax/Bcl-2 and the expression of cleaved caspase-3, which are proteins involved in apoptosis. The Bax/Bcl-2 ratio and cleaved caspase-3 expression in both cell lines significantly increased in the cotreatment group. In addition, immunocytochemistry results confirm that the expression of activated cas-3 was significantly increased in both cell lines in the cotreatment group. Regarding cell-cycle arrest, previous studies have reported that ERB-041 induced G1-phase arrest in human cancers by decreasing cyclin D1 expression, which regulates the cell-cycle transition from the G1 phase to S phase through PI3K/AKT pathway inhibition [34]. In contrast, genistein induces G2/M phase arrest through PI3K/AKT pathway inhibition in several human tumors, and cyclin D1 is upregulated [35,36]. Our cell-cycle-arrest assay results for CMT-U27 and CF41.Mg cells were very different. In CMT-U27 cells, there was an increase in CDK4 expression and a decrease in cyclin D1 expression, which synergistically induced G0/G1 phase arrest. In contrast, in CF41.Mg, the expression of CDK4 decreased and that of cyclin D1 increased, synergistically inducing G2/M phase arrest. We assumed that these contrasting results were due to differences in the characteristics of the two cell lines. In a previous study, it was reported that when human liver cancer cell lines HepG2 and Hep3B were treated with doxorubicin, the two cell lines responded differently to the drug, with HepG2 showing a G1 arrest and Hep3B showing a G2/M arrest [37]. These results are consistent with those of previous studies, and this study confirmed the same effect in both CMT cell lines. Based on these results, we conclude that ERB-041 and genistein synergistically exert anticancer effects by inducing apoptosis and cell-cycle arrest in CMT cells.

Next, we determined whether these anti-cancer effects of ERB-041 and genistein were mediated through reduced ERα expression and through the PI3K/AKT pathway. ERB-041 and genistein have been reported to reduce the expression of ERα in human tumors, and ERα has been reported to regulate apoptosis in breast cancer [38,39,40]. Furthermore, the expression of the PI3K/AKT pathway in breast cancer is ERα-dependent. In detail, the underlying mechanism involves ERα directly binding to PI3K, leading to an increase in PI3K expression and a decrease in the expression of PTEN, which functions in opposition to PI3K [41,42]. Our results showed that the expression of ERβ was synergistically reduced by ERB-041 and genistein in CMT-U27 and CF41.Mg cell lines despite treatment with ERβ agonists. Noteworthily, our experiments showed a significant decrease in the expression of ERα regardless of the decreased expression of ERβ, contrary to what is commonly known about ERβ inhibiting ERα [9]. In addition, Western blotting demonstrated that the expression of p-PI3K, p-AKT, and p-mTOR was synergistically reduced in both cell lines. The expression of PTEN, a negative regulator of PI3K, was reduced, despite the decreased expression of p-PI3K [43,44]. We focused on PTEN to understand the paradoxical expression of these proteins. The decreased expression of proteins involved in the PI3K/AKT pathway is speculated to be attributable to the reduced expression of ERα, leading to a diminished direct binding to PI3K. The expression of PTEN is decreased when PI3K expression is inhibited [45]. Furthermore, knockdown of PTEN induces a decrease in ERβ expression [46]. Based on the results reported in previous studies, we speculated that the direct effects of ERB-041 and genistein reduced the expression of ERα and PI3K, which in turn reduced the expression of PTEN, and that this would have led to a reduction in the expression of ERβ. Therefore, we determined that ERB-041 and genistein exert their anti-cancer effects by directly inhibiting the expression of ERα, which in turn downregulates the expression of the PI3K/AKT pathway in both CMT cells.

In conclusion, our results showed that treatment of two different CMT cells with ERβ agonist ERB-041 and genistein synergistically decreased the PI3K/AKT pathway expression by decreasing the expression of ERα. Thus, we suggest that cotreatment with ERB-041 and genistein has the potential to be effectively used in the treatment of CMTs.

## 4. Materials and Methods

### 4.1. Cell Culture and Reagents

CMT-U27 and CF41.Mg cell lines were purchased from the American Type Culture Collection (Manassas, VA, USA) and cultured in Roswell Park Memorial Institute 1640 medium (CMT-U27 cells; HyClone, Logan, UT, USA) or Dulbecco’s modified Eagle’s medium (CF41.Mg cells; Gibco, Grand Island, NY, USA) supplemented with 10% fetal bovine serum (FBS; Atlas Biologicals, Fort Collins, CO, USA), 100 unit/mL of penicillin (100 unit/mL) and 100 μg of streptomycin (Sigma-Aldrich, St. Louis, MO, USA). All cells were incubated at 37 °C in 5% CO_2_. ERB-041 and genistein were purchased from Sigma-Aldrich (ERB-041; PZ0183, Genistein; G6649).

### 4.2. MTS Assay

To assess the synergistic effect of palmatine on cell viability, we conducted a CellTiter 96^®^ AQueous One Solution Cell Proliferation Assay (Promega Corporation, Madison, WI, USA) based on the MTS assay. CMT-U27 cells were plated at 1 × 10^4^ cells and CF41.Mg cells were plated at 5 × 10^3^ cells in 96-well plates with 100 μL of medium and incubated at 37 °C for 24 h. Then, the cells were treated with ERB-041 and genistein for 24 h at each concentration. Following treatment, 20 μL of CellTiter 96^®^ AQueous One Solution Reagent was added to each well and incubated for 2 h at 37 °C. The absorbance was measured at 490 nm using a microplate reader (Spectramax M2; Molecular Devices, San Jose, CA, USA).

### 4.3. Annexin-V/PI Staining

Cell death in CMT-U27 and CF41.Mg cells was evaluated by flow cytometry using an Annexin-V assay (Santa Cruz Biotechnology, Inc., Dallas, TX, USA) according to the manufacturer’s protocol. Annexin-V content was estimated by measuring the fluorescence at 488 nm (excitation) and 525 nm (emission) using the Guava easyCyte HT system (Millipore, Billerica, MA, USA).

### 4.4. Western Blotting

CMT-U27 and CF41.Mg cells were lysed in cold lysis buffer supplemented with a protease inhibitor cocktail (Sigma-Aldrich). Protein extraction was followed by separation using sodium dodecyl sulfate-polyacrylamide gel electrophoresis and subsequent transfer to nitrocellulose membranes. To inhibit non-specific binding, the membranes were treated with 5% skim milk and then incubated overnight at 4 °C with the following primary antibodies in a blocking buffer: rabbit polyclonal anti-ERβ (ab3576; 1:1000; Abcam, Cambridge, MA, USA), mouse monoclonal anti-ERα (MA1-12692; 1:1000; Invitrogen, Carlsbad, CA, USA), rabbit polyclonal anti-p-PI3K (AF3242; 1:1000; Affinity biosciences, Cincinnati, OH, USA), rabbit polyclonal anti-AKT (9272; 1:1000; Cell Signaling Technology, Inc., Beverly, MA, USA), rabbit polyclonal anti-p-AKT (9271; 1:1000; Cell Signaling Technology), rabbit monoclonal anti-mTOR (2983; 1:1000; Cell Signaling Technology), rabbit monoclonal anti-p-mTOR (5536; 1:1000; Cell Signaling Technology), rabbit polyclonal anti-PTEN (bs0686-R; 1:1000; Bioss, Inc., Beijing, China), mouse monoclonal anti-p-PTEN (sc-377573; 1:1000; Santa Cruz Biotechnology, Santa Cruz, CA, USA), mouse monoclonal anti-Bcl-2 (Sc-7382; 1:1000; Santa Cruz Biotechnology), rabbit polyclonal anti-Bax (2772; 1:1000; Cell Signaling Technology), rabbit polyclonal anti-caspase-3 (9662; 1:1000; Cell Signaling Technology), mouse monoclonal anti-Cyclin D1 (AHF0082; 1:1000; Invitrogen), rabbit polyclonal anti-CDK4 (11026-1-AP; 1:1000; Proteintech, Chicago, IL, USA) and mouse monoclonal anti-β-actin (A5441; 1:1000; Sigma-Aldrich). Membranes were then incubated with horseradish peroxidase (HRP)-conjugated secondary antibodies for 1 h at room temperature. Chemiluminescent signals were amplified using WESTSAVE Gold (LF-QC0103; Abfrontier, Seoul, Republic of Korea) or WESTSAVE Star (LF-QC0106; Abfrontier) and subsequently captured using a Fusion FX7 acquisition system (Vilbert Lourmat, Eberhardzell, Germany). Band densities were quantified using Quantity One (version 4.6.6) and normalized to that of β-actin. The intensity was then presented relative to that of the control.

### 4.5. Cell-Cycle-Arrest Assay

CMT-U27 and CF41.Mg cells were seeded in 6-well plates at 5 × 10^5^ and 25 × 10^4^ cells, respectively. After treatment, cells were fixed overnight in ice-cold ethanol/PBS (7:3). The cells were then washed twice with PBS and incubated with FxCycle™ PI/RNase Staining Solution (F10797; Invitrogen) for 30 min at room temperature. The cells were analyzed using a CytoFLEX S V4-B2-Y0-R3 Flow Cytometer (C02948; Beckman Coulter, Brea, CA, USA) and the data were analyzed using CytExpert software (version 2.5.0.77).

### 4.6. Immunocytochemistry

CMT-U27 and CF41.Mg cells were cultured on gelatin-coated coverslips. Cells were fixed with 2% paraformaldehyde for 20 min at 4 °C and permeabilization using 0.5% Triton X-100 in PBS for 10 min. Blocking was performed using 5% animal serum (donkey or goat) in 2% bovine serum albumin in PBS for 1 h at room temperature. Subsequently, the cells were treated with primary antibodies against anti-ERβ (1:200; Abcam), anti-ERα (1:200; Invitrogen), anti-p-PI3K (1:200; Affinity Biosciences), and anti-PTEN (1:200; Bioss) in a blocking solution at 4 °C overnight. The cells were incubated with Alexa Fluor^TM^ 594 conjugated goat anti-mouse IgG (A-11005; 1:1000; Invitrogen) or Alexa Fluor^®^ 488 conjugated goat anti-rabbit IgG (ab150077; 1:1000; Abcam). The cell nuclei were counterstained with 4′,6-diamidino-2-phenylindole (DAPI). The cells were then mounted using mounting medium (Dako, Carpinteria, CA, USA) and images were captured using a THUNDER Imager 3D Live Cell & 3D Cell Culture System (Leika Microsystems, Wetzlar, Germany). The mean fluorescence intensity for each channel in the three distinct regions was quantified using ImageJ software (version 1.52a). The fluorescence intensity was expressed relative to that of the control.

### 4.7. Statistical Analysis

All data are presented as mean ± standard deviation (SD). Statistical significance between groups was determined using an unpaired Student’s *t*-test. One-way analysis of variance (ANOVA), followed by Bonferroni post hoc tests, was conducted to determine significant differences among multiple groups. Statistical analyses were performed using GraphPad Prism software (version 5.0). Statistical significance was set at *p* < 0.05.

## Data Availability

All data generated or analyzed during this study are included in this published article.

## Supplementary Materials

The supporting information can be downloaded at: https://www.mdpi.com/article/10.3390/ijms25052466/s1.
