# Supplementary material for: Synergistic Anti-Cancer Effects of ERB-041 and Genistein through Estrogen Receptor Suppression-Mediated PI3K/AKT Pathway Downregulation in Canine Mammary Gland Tumor Cells
Source: Int J Mol Sci. 2024 Feb 20;25(5):2466. doi: 10.3390/ijms25052466 (PMC10931264; doi:10.3390/ijms25052466)
Supplement: Supplementary file 1 [file ijms-25-02466-s001.zip › ijms-2867341-supplementary.pdf]

Supplementary Figure S1

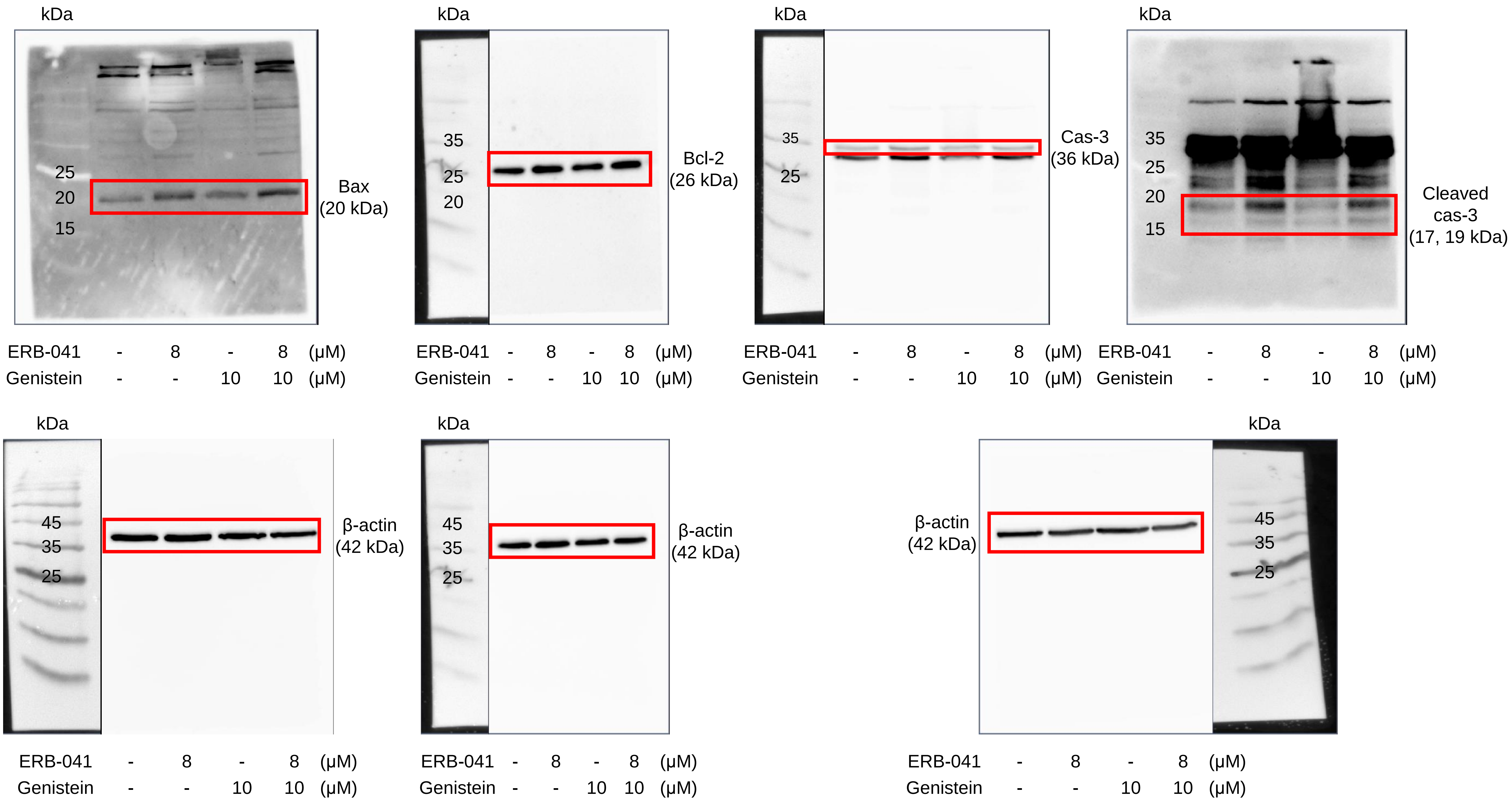

Supplementary Figure S1

Original, uncropped western blot images for Figure 3A, B. The bands of Bax, Bcl-2, caspase-3, and cleaved caspase-3, and molecular weights of markers in CMT-U27 cell.

Supplementary Figure S2

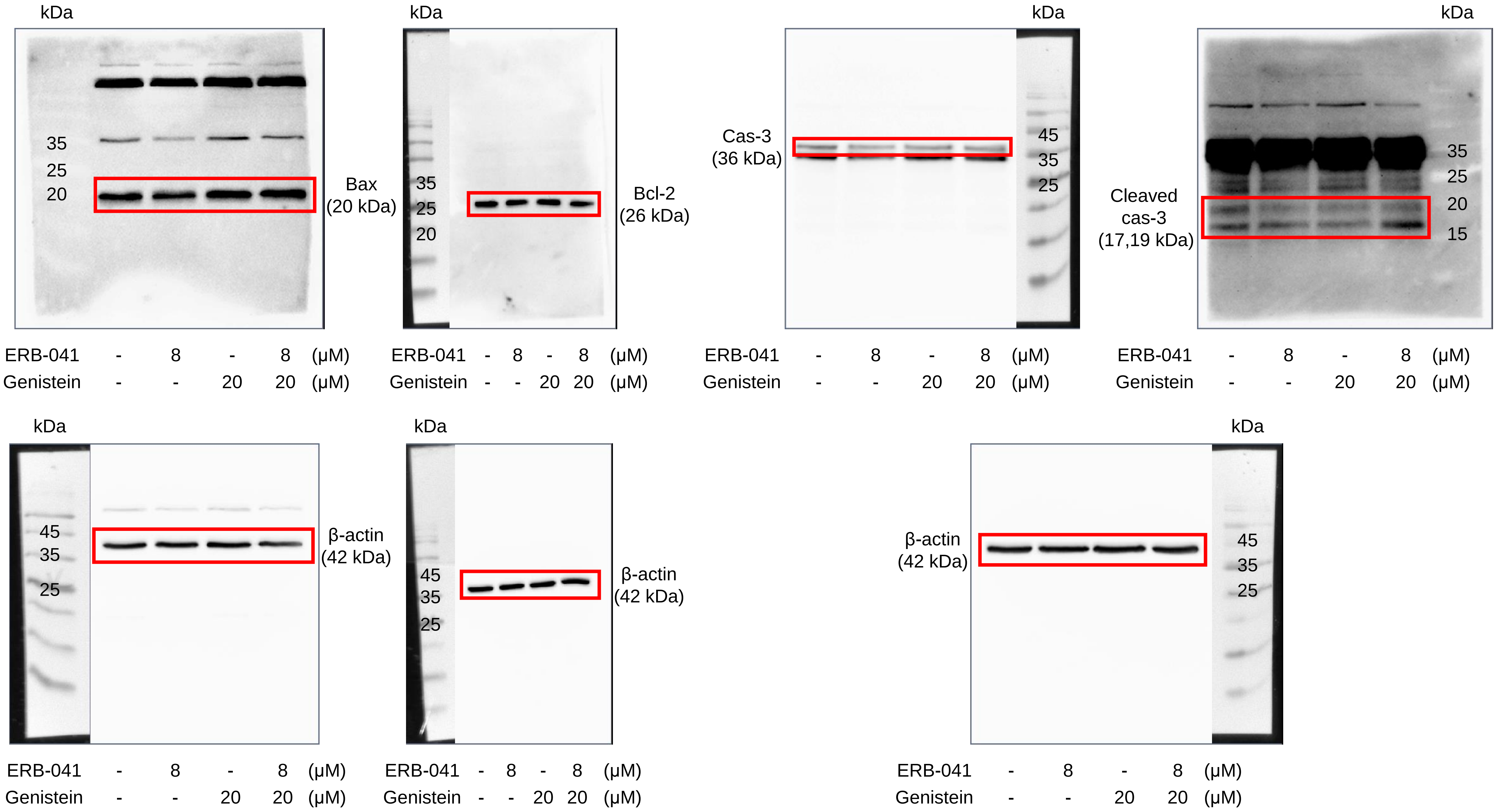

Supplementary Figure S2

Original, uncropped western blot images for Figure 3C, D. The bands of Bax, Bcl-2, caspase-3, and cleaved caspase-3, and molecular weights of markers in CF41.mg cell.

Supplementary Figure S3

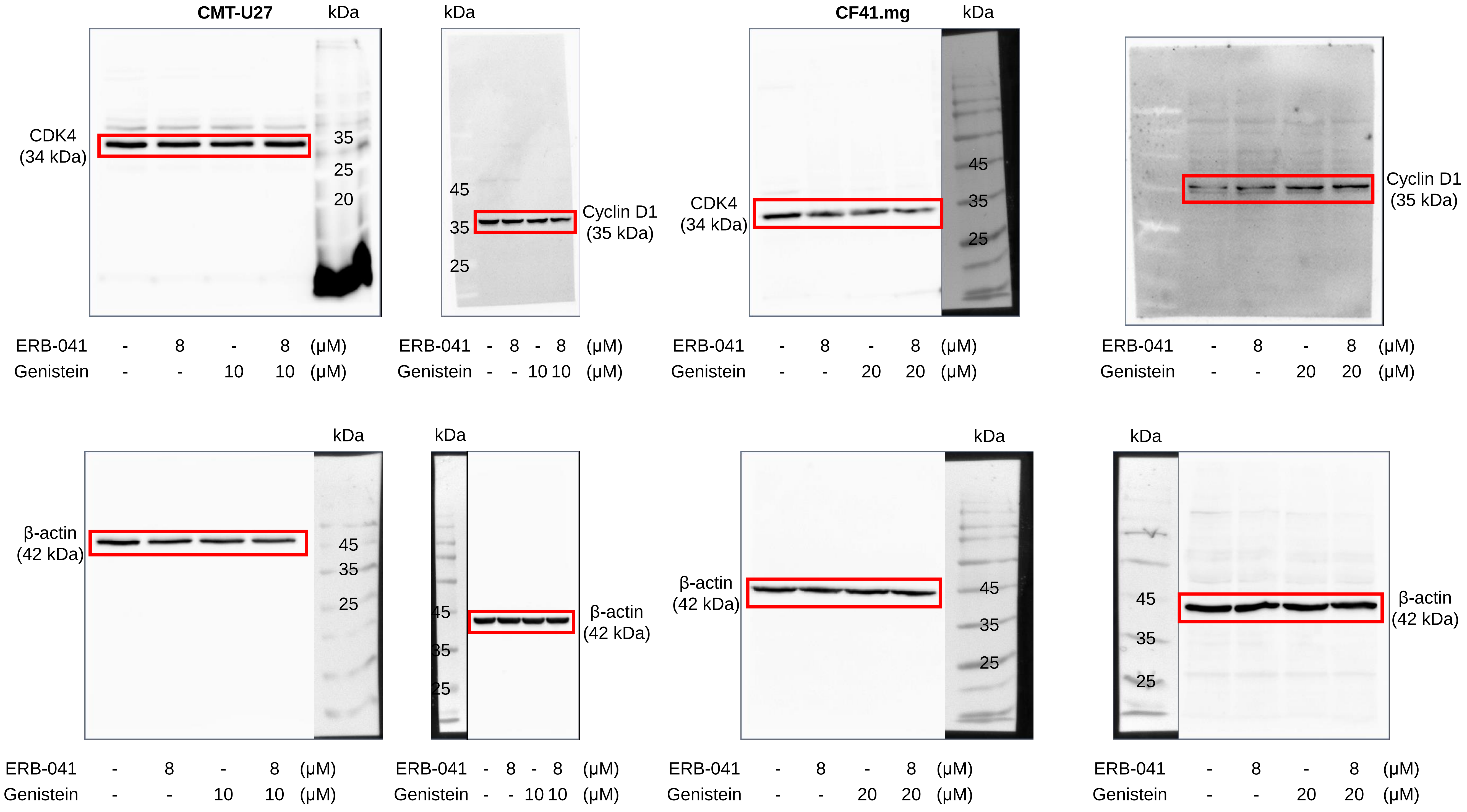

Supplementary Figure S3

Original, uncropped western blot images for Figure 4. The bands of CDK4 and cyclin D1, and molecular weights of markers in CMT-U27 and CF41.mg cells.

Supplementary Figure S4

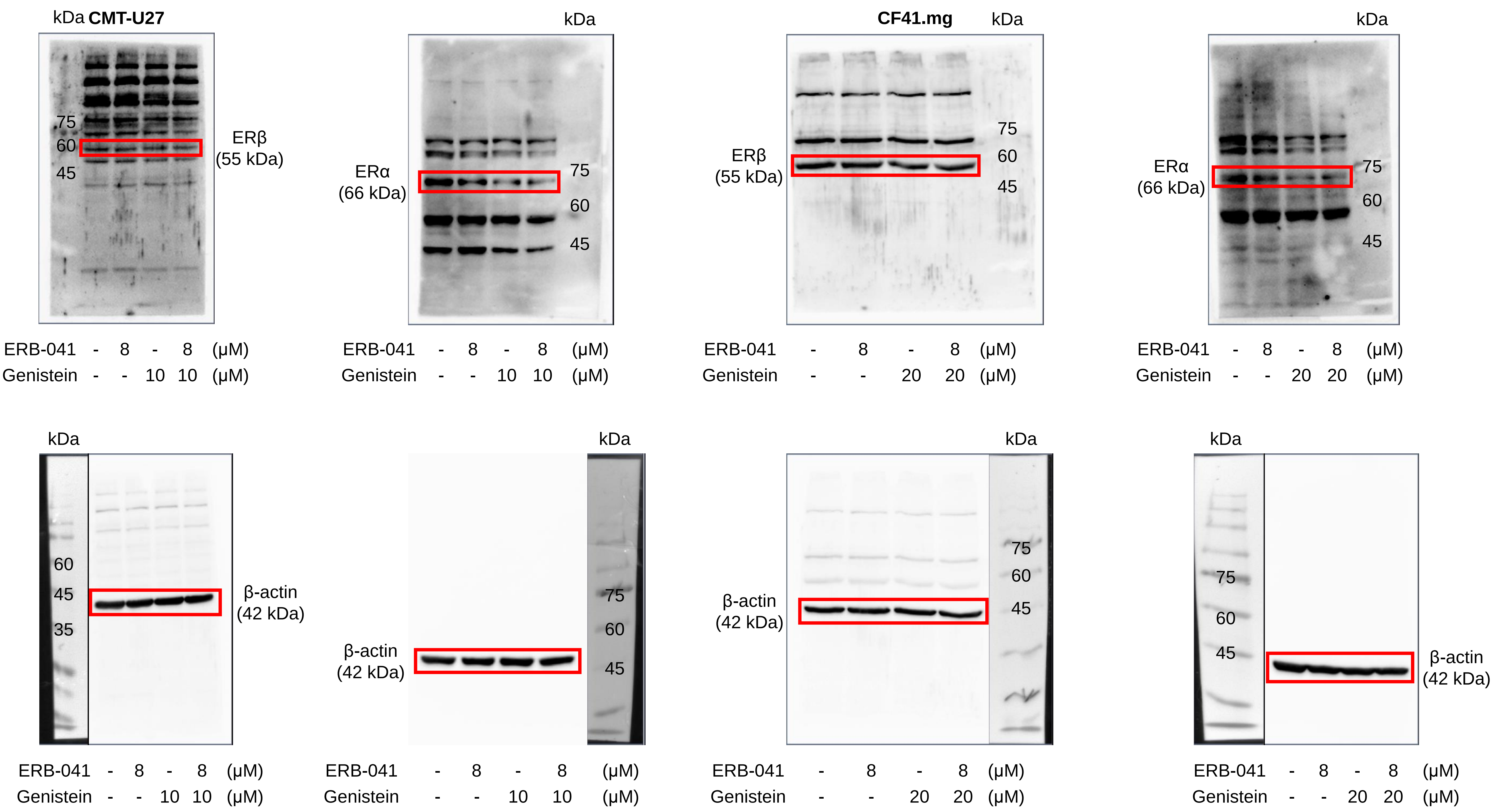

Supplementary Figure S4

Original, uncropped western blot images for Figure 5. The bands of ERβ and ERα, and molecular weights of markers in CMT-U27 and CF41.mg cells.

Supplementary Figure S5

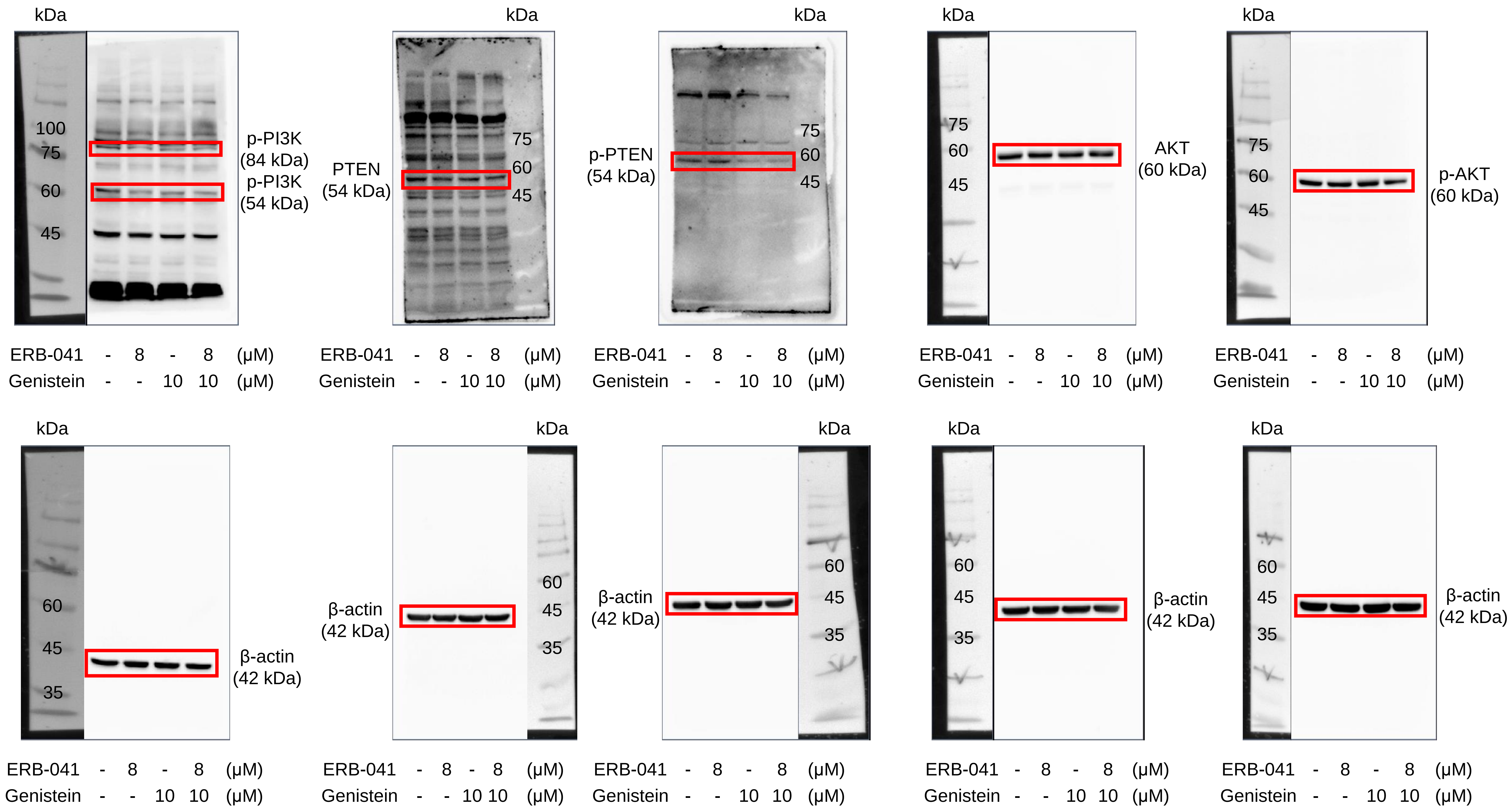

Supplementary Figure S5

Original, uncropped western blot images for Figure 6A-C. The bands of p-PI3K, PTEN, p-PTEN, AKT and p-AKT, and molecular weights of markers in CMT-U27 cell.

Supplementary Figure S6

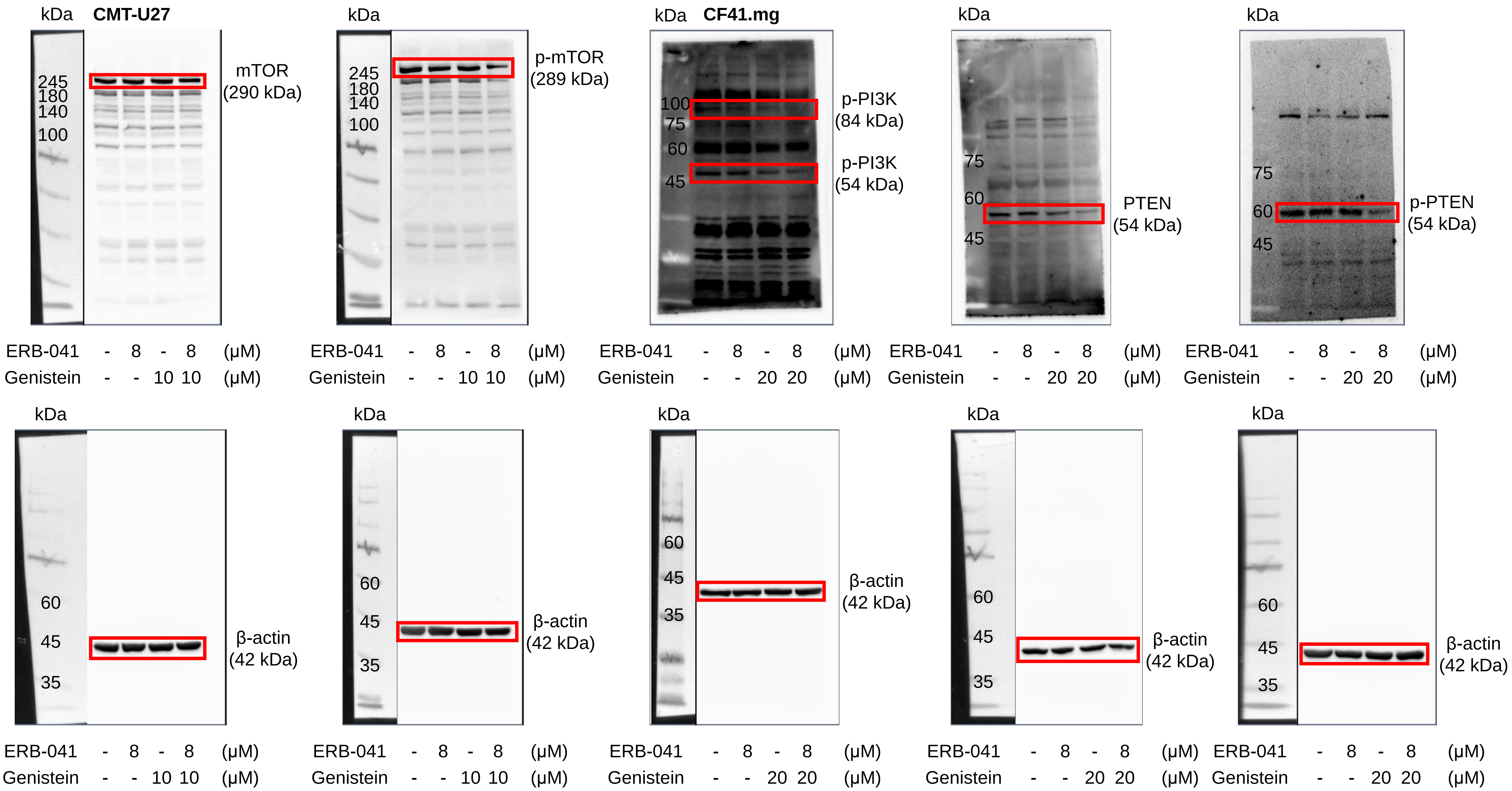

Supplementary Figure S6

Original, uncropped western blot images for Figure 6D-F. The bands of mTOR, p-mTOR, p-PI3K, PTEN and p-PTEN, and molecular weights of markers in CMT-U27 cell.

Supplementary Figure S7

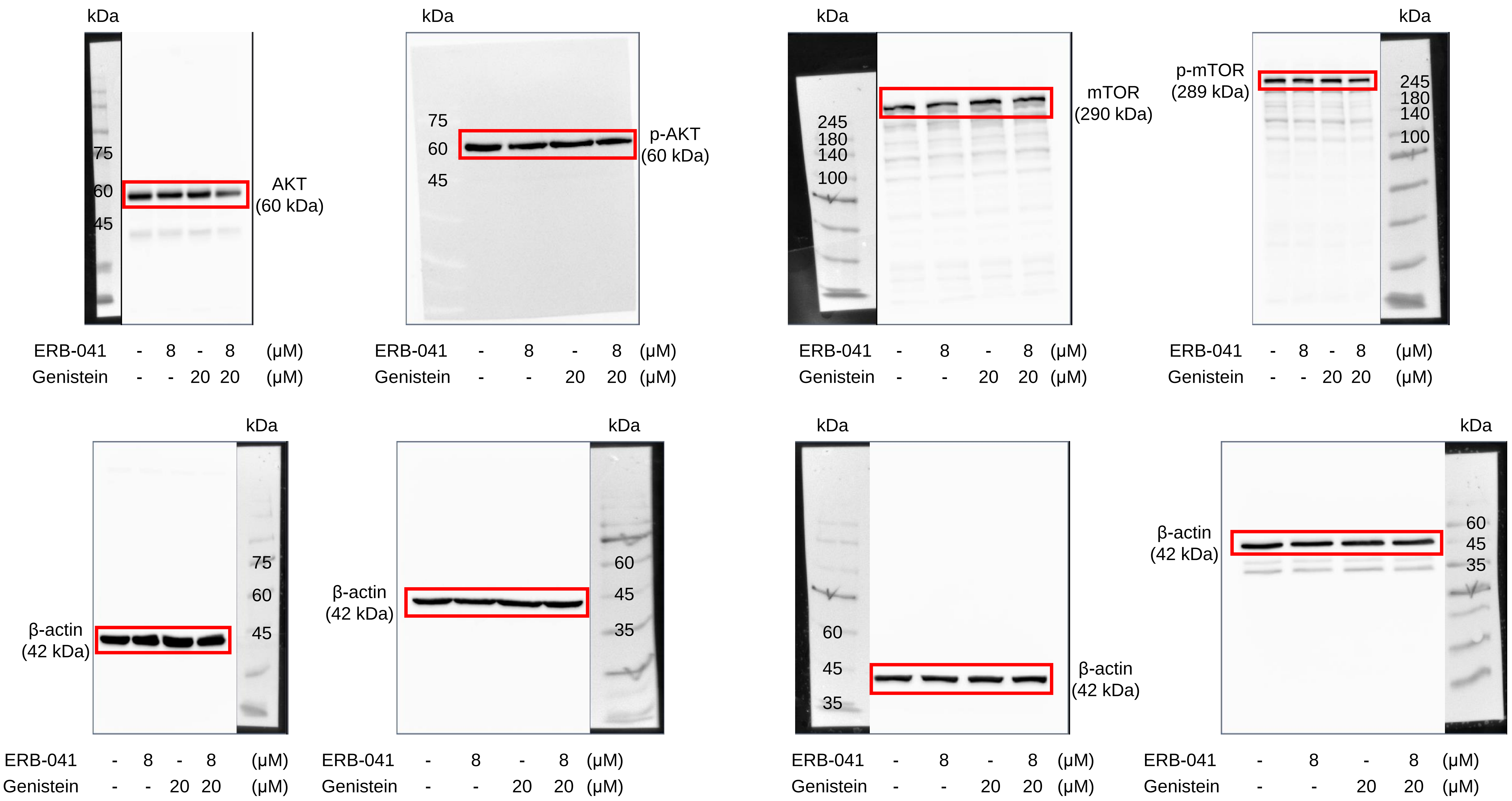

Supplementary Figure S7

Original, uncropped western blot images for Figure 6G, H. The bands of AKT, p-AKT, mTOR and p-mTOR, and molecular weights of markers in CF41.mg cell.
